# Risk Factors for Esophageal Collateral Veins in Cirrhosis with and without Previous Endoscopic Esophageal Variceal Therapy

**DOI:** 10.1155/2022/6666791

**Published:** 2022-01-04

**Authors:** Qianqian Li, Xiaozhong Guo, Ji Feng, Xiangbo Xu, Saurabh Chawla, Hongyu Li, Xingshun Qi

**Affiliations:** ^1^Department of Gastroenterology, General Hospital of Northern Theater Command, Shenyang 110840, China; ^2^Postgraduate College, Dalian Medical University, Dalian 116044, China; ^3^Postgraduate College, Shenyang Pharmaceutical University, Shenyang 110840, China; ^4^Department of Medicine, Division of Digestive Diseases, Emory University School of Medicine, Atlanta, GA, USA

## Abstract

**Background:**

Portosystemic collateral vessels are a sign of portal hypertension in liver cirrhosis. Esophageal collateral veins (ECVs) are one major type of portosystemic collateral vessels, which increase the recurrence of esophageal varices and bleeding after variceal eradication. However, the risk factors for ECVs were still unclear.

**Methods:**

We retrospectively screened cirrhotic patients who had contrast-enhanced computed tomography (CT) images to evaluate ECVs and upper gastrointestinal endoscopic reports to evaluate gastroesophageal varices at our department. Univariate and multivariate logistic regression analyses were performed to explore the independent risk factors for ECVs. Odds ratios (ORs) were calculated. Subgroup analyses were performed in patients with and without previous endoscopic variceal therapy which primarily included endoscopic variceal ligation (EVL) and endoscopic injection sclerotherapy (EIS).

**Results:**

Overall, 243 patients were included, in whom the prevalence of ECVs was 53.9%. The independent risk factors for ECVs were hepatitis C virus infection (OR = 0.250, *p* = 0.026), previous EVL (OR = 1.929, *p* = 0.044), platelet (OR = 0.993, *p* = 0.008), and esophageal varices needing treatment (EVNTs) (OR = 2.422, *p* = 0.006). The prevalence of ECVs was 60.8% (73/120) in patients undergoing EVL, 50% (10/20) in those undergoing EIS, and 47.5% (48/101) in those without previous endoscopic variceal therapy. The independent risk factors for ECVs were the use of nonselective beta-blockers (OR = 0.294, *p* = 0.042) and EVNTs (OR = 3.714, *p* = 0.006) in subgroup analyses of patients with and without previous endoscopic variceal therapy, respectively.

**Conclusions:**

The presence of ECVs should be closely associated with the severity of portal hypertension in liver cirrhosis. Risk of ECVs might be increased by previous EVL.

## 1. Introduction

Esophageal varices (EVs) are the most common collaterals in advanced cirrhosis that are located inside the esophageal lumen [1]. Bleeding from EVs remarkably increases the risk of mortality [2, 3]. The recommendations on management of EVs bleeding are clearly given by the current practice guidelines and consensus, and the most commonly recommended approach is endoscopic variceal therapy, such as endoscopic variceal ligation (EVL) and endoscopic injection sclerotherapy (EIS) [3, 4].

Esophageal collateral veins (ECVs) refer to the portosystemic collateral vessels outside the esophageal lumen [5]. Individual studies and meta-analyses by our and other teams suggest a high prevalence of ECVs in patients with portal hypertension and a remarkable impact of ECVs on the recurrence of EVs [6–9]. However, the risk factors for developing ECVs remained unclear. On the other hand, it seems that the incidence of ECVs would be different between patients who underwent EVL and EIS [10, 11]. In the present study, we aimed to explore the risk factors for developing ECVs in cirrhotic patients with and without endoscopic esophageal variceal therapy.

## 2. Materials and Methods

### 2.1. Patients

In this single-center retrospective study, we screened cirrhotic patients who underwent both contrast-enhanced computed tomography (CT) and upper gastrointestinal endoscopy between December 2014 and May 2019. The data were derived from our prospectively established database collecting cirrhotic patients admitted to our department. This study was approved by the medical ethical committee of our hospital, and the approval number was *k* (2019) 35.

The inclusion criteria were as follows: (1) patients were diagnosed with cirrhosis according to the medical history, clinical features, laboratory, and/or imaging results and (2) both endoscopic examinations and contrast-enhanced CT scans were performed at their admissions. Patients whose contrast-enhanced CT images were not well preserved were excluded.

### 2.2. Data Collection

We collected the data as follows: age, sex, etiology of liver diseases, hepatic encephalopathy (HE), gastrointestinal bleeding (GIB), ascites, history of GIB, history of endoscopic variceal therapy, endoscopic variceal therapy approaches including EVL and EIS, interval between previous endoscopic variceal therapy and present contrast-enhanced CT scans, red blood cell, hemoglobin, white blood cell (WBC), platelet (PLT), total bilirubin (TBIL), albumin (ALB), alanine aminotransferase, aspartate aminotransferase, alkaline phosphatase (AKP), *γγ*-glutamine transferase (GGT), blood urea nitrogen, creatinine (Cr), sodium, potassium, prothrombin time (PT), activated partial thromboplastin time, and international normalized ratio (INR). EVL was performed by ligation device MBL-6-F (Wilson-Cook Medical Inc., NC, USA). The drug used during EIS was polycinnamyl alcohol (Shaanxi TIANYU Pharmaceutical Co., Ltd., Shaanxi, China). We also recorded the use of nonselective beta-blockers (NSBBs) within 1 month before admission. The Child-Pugh and model for end stage of liver disease (MELD) scores were calculated as follows [12, 13].

Child-Pugh score = ALB score + TBIL score + INR score + ascites score + HE score. MELD score = 9.57 × ln [Cr (*µ*mol/L) × 0.011] + 3.78 × ln [TBIL (*µ*mol/L) × 0.058] + 11.2 × ln(INR) + 6.43.

### 2.3. Evaluation of ECVs on Contrast-Enhanced CT

**Figure 1:**
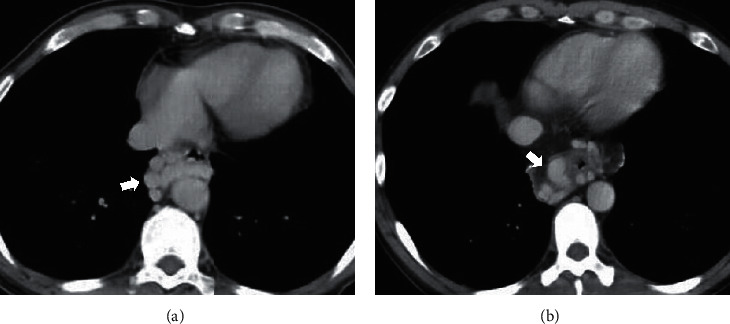
Esophageal collateral veins on contrast-enhanced CT scans.

The presence of ECVs on contrast-enhanced CT images was evaluated by two observers (QL and XQ). We were blind to endoscopic findings before analyzing the CT images. ECVs were defined as enhanced dilated vascular shadow surrounding the esophagus at the portal vein phases of contrast-enhanced CT images (Figure 1).

### 2.4. Evaluation of EVs on Endoscopy

As for the patients who underwent endoscopic variceal therapy, the endoscopic findings regarding EVs would be extracted, if endoscopic examinations were performed after contrast-enhanced CT scans during the same hospitalizations; by contrast, the endoscopic findings would not be extracted, if endoscopic examinations were performed before contrast-enhanced CT scans during the same hospitalizations. As for the patients who did not undergo endoscopic variceal therapy, the endoscopic findings regarding EVs would be extracted regardless of the order of contrast-enhanced CT and endoscopy. EVs needing treatment (EVNTs) include moderate and severe EVs that are diagnosed according to the Chinese consensus regarding management of gastroesophageal varices [4]. In details, they were defined as follows: (1) straight or slightly tortuous EVs with red color (RC) signs; (2) serpentine tortuous uplifted EVs with RC signs with or without RC signs; or (3) beaded, nodular, or tumor-like EVs with or without RC signs.

### 2.5. Statistical Analyses

Continuous variables were expressed as mean ± standard deviation and median (range) and compared by using Mann–Whitney *U* test. Categorical variables were expressed as frequencies and percentages and compared by Chi-square tests. A two-sided *p* < 0.05 was considered statistically significant. Univariate and multivariate logistic regression analyses were used to identify the risk factors of developing ECVs. Variables with a *p* < 0.1 in univariate analysis were included in multivariate analyses. Only one of the variables with collinearity was selected in multivariate analyses. Odds ratios (ORs) and 95% confidence intervals (CIs) were calculated. Subgroup analyses were performed in patients with and without history of endoscopic variceal therapy. Statistical analyses were performed using the SPSS software version 20.0 (IBM Corp, Armonk, NY, USA).

## 3. Results

### 3.1. Patients

A total of 243 cirrhotic patients who underwent contrast-enhanced CT and upper gastrointestinal endoscopy at the same hospitalization were included (Table 1). Among them, 71.2% (173/243) of patients were male and 28.8% (70/243) were female. Hepatitis B virus infection and alcohol abuse were the major etiologies. One hundred and forty-one patients underwent endoscopic variceal therapy, of whom 121 and 20 underwent EVL and EIS as the last endoscopic variceal therapeutic approach, respectively. The interval between last endoscopic variceal therapy and CT could not be calculated in 4 patients due to the lack of specific date. The information regarding use of NSBBs was available in 192 patients, of whom 16.1% (31/192) took NSBBs within 1 month before admission. The prevalence of ECVs on contrast-enhanced CT scans was 53.9% (131/243).

### 3.2. Overall Comparison between ECVs and No ECVs Groups

Patients with ECVs had significantly lower proportion of hepatitis C virus (HCV) infection (*p* = 0.011) and levels of WBC (*p* < 0.0001), PLT (*p* < 0.0001), AKP (*p* = 0.014), and GGT (*p* = 0.042) and higher proportions of EVs (*p* < 0.0001), EVNTs (*p* = 0.002), and previous EVL (*p* = 0.044) and levels of INR (*p* = 0.005), PT (*p* = 0.005), and MELD score (*p* = 0.017) than those without ECVs (Table 2).

Prevalence of ECVs in patients who underwent EVL and EIS and those who did not undergo endoscopic variceal therapy was 60.8%, 50%, and 47.5%, respectively.

Univariate logistic regression analyses showed that HCV, previous EVL, WBC, PLT, ALT, AST, AKP, MELD score, EVs, and EVNTs were significantly associated with ECVs. Because there is a collinearity between WBC and PLT, only PLT was included in the multivariate analyses. Because there is a collinearity between ALT and AST, only ALT was included in the multivariate analyses. Because there is a collinearity between EVs and EVNTs, only EVNTs were included in the multivariate analyses. Multivariate logistic regression analyses showed that HCV (OR = 0.250, 95% CI = 0.074–0.846, *p* = 0.026), previous EVL (OR = 1.929, 95% CI = 1.016–3.661, *p* = 0.044), PLT (OR = 0.993, 95% CI = 0.988–0.998, *p* = 0.008), and EVNTs (OR = 2.422, 95% CI = 1.297–4.522, *p* = 0.006) were independently associated with ECVs (Table 3).

### 3.3. Subgroup Analysis in Patients Who Underwent Endoscopic Variceal Therapy

Compared with those without ECVs, patients with ECVs had significantly lower proportion of HCV (*p* = 0.010) and levels of WBC (*p* = 0.036), PLT (*p* = 0.014), AST (*p* = 0.045), AKP (*p* < 0.0001), and GGT (*p* = 0.008) and higher levels of Cr (*p* = 0.010) and MELD score (*p* = 0.018) (Table 4).

Univariate logistic regression analyses showed that sex, HCV, history of GIB, NSBBs, WBC, PLT, AKP, Cr, MELD score, and EVNTs were significantly associated with ECVs. Because there is a collinearity between WBC and PLT, only PLT was included in the multivariate analyses. Because there is a collinearity between Cr and MELD score, only MELD score was included in the multivariate analyses (Table 5). Multivariate logistic regression analyses showed that the use of NSBBs (OR = 0.294, 95% CI = 0.091–0.957, *p* = 0.042) was independently associated with ECVs (Table 5).

### 3.4. Subgroup Analysis in Patients Who Did Not Undergo Endoscopic Variceal Therapy

Compared with those without ECVs, patients with ECVs had significantly lower levels of WBC (*p* = 0.002) and PLT (*p* = 0.003) and higher proportions of EVs (*p* < 0.0001) and EVNTs (*p* = 0.002) and levels of INR (*p* = 0.007) and PT (*p* = 0.010) (Table 6).

Univariate logistic regression analyses showed that ascites, PLT, EVs, and EVNTs were significantly associated with ECVs. Because there is a collinearity between EVs and EVNTs, only EVNTs were included in the multivariate analyses (Table 7). Multivariate logistic regression analyses showed that the presence of EVNTs (OR = 3.714, 95% CI = 1.469–9.391, *p* = 0.006) was independently associated with ECVs (Table 7).

## 4. Discussion

The present study showed that HCV infection, a low PLT count, presence of EVNTs, and previous EVL were independently associated with ECVs in cirrhosis.

EVL and EIS were the common endoscopic variceal therapy approaches for controlling variceal hemorrhage and preventing from first or recurrent bleeding from high-risk varices. Current guidelines recommend EVL as the preferred endoscopic therapy because EVL may be superior to EIS in terms of complications and patients' outcomes [14, 15]. In details, a meta-analysis showed that patients who underwent EVL might have significantly higher variceal elimination rate and lower rebleeding rate than those who underwent EIS [16]. However, the choice of endoscopic variceal therapy might influence the presence of ECVs [8, 10]. The present study also reported that the prevalence of ECVs was different between patients with and without history of endoscopic esophageal variceal therapy, and it was higher in patients who underwent EVL than those who underwent EIS (60.8% versus 50%). This could be explained that EVL only achieved superficial eradication of EVs through a mechanical constriction, but EIS could act on submucosal tissues through a chemical reaction, which would reduce the number and size of ECVs and even obliterate ECVs completely [11, 17, 18].

NSBBs are recommended as another first-line therapy for preventing variceal bleeding in patients with high-risk varices because it could significantly reduce portal pressure [15, 19]. Besides, a previous study has confirmed that NSBBs could slow the development of ECVs and reduce the size of ECVs [20]. Similarly, the present study demonstrated that patients with history of endoscopic variceal therapy who adhered to the use of NSBBs had a lower risk of developing ECVs.

EVs and ECVs, the common types of portosystemic collateral veins, were one of the common consequences of portal hypertension [1, 21]. According to their location with the esophagus, ECVs can be classified as para-esophageal veins (para-EVs), peri-esophageal veins (peri-EVs), and perforating veins (PVs) [22]. Endoscopic color Doppler ultrasonography demonstrates a blood flow communication between para-EVs or peri-EVs and EVs through PVs [23]. Therefore, it is readily understood that EVNTs are more likely to be accompanied with ECVs. On the other hand, a low PLT count has been widely considered as an indicator for severity of hypersplenism and portal hypertension in cirrhosis [24]. In details, PLT count is a major component of PLT count to spleen diameter ratio (PSR) [25] and Baveno VI criteria [15, 26], which are two important indexes for evaluating EVNTs. The present study also found that a low PLT count was associated with ECVs, which further suggested that the presence of ECVs should be in parallel with the severity of portal hypertension.

Most previous studies have suggested that the presence of ECVs may be associated with the recurrence of EVs [9]. However, it is possible that portal pressure can be reduced by ECVs as collateral vessels. Especially if para-ECVs were not connected with esophageal varices through PVs, they would decrease the risk of variceal recurrence after endoscopic variceal therapy [27]. Therefore, the association of ECVs with recurrence of EVs should be further explored.

There were several limitations in our study. First, this was a single-center retrospective study, in which selection bias and data missing were inevitable. Second, we employed CT scans, but not endoscopic ultrasonography. Thus, the types of ECVs could not be accurately classified. Third, CT images are not ideal in a few patients, in whom ECVs and EVs were not clearly distinguished on CT scans. Fourth, ECVs mostly appear as irregular blood vessel clusters on CT scans, so we cannot measure the diameter of ECVs and record the changes of ECVs.

In conclusion, the presence of ECVs was closely associated with the severity of portal hypertension indicated by lower PLT count and EVNTs. Additionally, EVL might induce the development of ECVs; by comparison, EIS might be more effective for eliminating ECVs. Further prospective studies should be needed to confirm this finding.

## Figures and Tables

**Table 1:** Characteristics of patients.

Variables	No. Pts	Mean ± SD Median (range) or frequency (percentage)
Age (years)	243	55.11 ± 10.39
Sex (male)	243	173 (71.2%)

Etiology of liver diseases		
Hepatitis B virus infection	243	103 (42.4%)
Hepatitis C virus infection	243	19 (7.8%)
Alcohol abuse	243	92 (37.9%)
Drug related	243	21 (8.6%)
Autoimmune liver diseases	243	17 (7.0%)

Clinical presentations at admission		
Hepatic encephalopathy	243	5 (2.1%)
Gastrointestinal bleeding	243	95 (39.1%)
Ascites (no/mild/moderate-severe)	243	112 (46.1%)/89 (36.6%)/42 (17.3%)

History		
History of gastrointestinal bleeding	243	161 (66.3%)
History of endoscopic variceal therapy	243	141 (58.0%)
EVL as last endoscopic variceal therapeutic approach	243	121 (49.8%)
EIS as last endoscopic variceal therapeutic approach	243	20 (8.2%)
Interval between last endoscopic variceal therapy and CT (days)	137	313.60 ± 371.29 190.00 (1.00–1676.00)
NSBBs within 1 month before admission	192	31 (16.1%)

Laboratory data		
Red blood cell (10^12^/L)	243	3.63 ± 0.90 3.73 (1.51–9.92)
Hemoglobin (g/L)	243	102.97 ± 28.41 103.00 (28.00–181.00)
White blood cell (10^9^/L)	243	4.22 ± 3.00 3.40 (0.70–21.60)
Platelet (10^9^/L)	243	102.79 ± 82.54 79.00 (15.00–681.00)
Total bilirubin (*µ*mol/L)	243	26.20 ± 26.62 17.90 (5.60–216.50)
Albumin (g/L)	243	34.67 ± 6.21 35.20 (14.20–71.40)
Alanine aminotransferase (U/L)	243	29.57 ± 28.99 22.14 (4.23–332.50)
Aspartate aminotransferase (U/L)	243	41.28 ± 34.30 30.78 (9.63–376.35)
Alkaline phosphatase (U/L)	243	111.77 ± 88.64 90.06 (24.35–983.93)
*γ*-Glutamyl transpeptidase (U/L)	243	91.81 ± 213.01 33.93 (7.49–1779.18)
Blood urea nitrogen (mmol/L)	243	5.72 ± 2.60 5.23 (1.86–20.15)
Creatinine (*µ*mol/L)	243	64.27 ± 17.27 61.71 (27.95–178.55)
Potassium (mmol/L)	243	3.89 ± 0.42 3.91 (2.42–5.87)
Sodium (mmol/L)	243	138.79 ± 3.11 139.10 (118.00–147.70)
Prothrombin time (seconds)	243	16.31 ± 2.50 15.80 (12.50–28.00)
Activated partial thromboplastin time (seconds)	243	40.66 ± 5.54 40.10 (19.80–71.30)
International normalized ratio	243	1.33 ± 0.28 1.27 (0.94–2.77)
Child-Pugh score	243	6.72 ± 1.65 6.00 (5.00–13.00)
Child-Pugh class (A/B/C)	243	133 (54.7%)/93 (38.3%)/17 (7.0%)
MELD score	243	6.46 ± 4.48 5.58 (−2.13–27.42)
EVs on endoscopy (no/yes/unknown)	211^a^	34 (16.1%)/177 (72.8%) 0 (0.0%)
EVNTs on endoscopy (no/yes/unknown)	211^a^	104 (49.3%)/106 (50.2%)/1 (0.5%)^b^
ECVs on CT (no/yes/unknown)	243	110 (45.3%)/131 (53.9%)/2 (0.8%)^c^

**Notes:** ^a^As for the patients who underwent endoscopic variceal therapy, only EVs on endoscopy performed after CT during the same hospitalizations were evaluated; as for the patients who did not undergo endoscopic variceal therapy, EVs on endoscopy performed during the same hospitalizations were evaluated, regardless of the order of CT and endoscopy; ^b^EVNTs could not be evaluated due to the absence of detailed grade of EVs in their endoscopic reports; ^c^ECVs could not be evaluated because the venous vessels were not obviously enhanced. Pts, patients; SD, standard deviation; EVL, endoscopic variceal ligation; EIS, endoscopic injection sclerotherapy; CT, computed tomography; NSBBs, nonselective beta-blockers; MELD, model for end stage of liver disease; EVs, esophageal varices; ECVs, esophageal collateral veins.

**Table 2:** Comparison between ECVs and no ECVs groups.

Variables	ECVs		No ECVs		*p* value
	No. Pts^a^	Mean ± SD Median (range) or frequency (percentage)	No. Pts^a^	Mean ± SD Median (range) or frequency (percentage)	
Age (years)	131	55.60 ± 10.78	110	54.55 ± 10.00	0.475
Sex (male/female)	131	96 (73.3%)/35 (26.7%)	110	76 (69.1%)/34 (30.9%)	0.473

Etiology of liver diseases					
Hepatitis B virus infection	131	61 (46.6%)	110	42 (38.2%)	0.190
Hepatitis C virus infection	131	5 (3.8%)	110	14 (12.7%)	**0.011**
Alcohol abuse	131	52 (39.7%)	110	40 (36.4%)	0.596
Drug related	131	11 (8.4%)	110	9 (8.2%)	0.952
Autoimmune liver diseases	131	7 (5.3%)	110	9 (8.2%)	0.378

Clinical presentations at admission					
Hepatic encephalopathy	131	1 (0.8%)	110	4 (3.6%)	0.269
Gastrointestinal bleeding	131	56 (42.7%)	110	38 (34.5%)	0.193
Ascites (no/mild/moderate-severe)	131	54 (41.2%)/52 (39.7%)/25 (19.1%)	110	56 (50.9%)/37 (33.6%)/17 (15.5%)	0.321

History					
History of gastrointestinal bleeding	131	89 (67.9%)	110	71 (64.5%)	0.579
History of endoscopic variceal therapy	131	83 (63.4%)	110	57 (51.8%)	0.071
EVL as last endoscopic variceal therapeutic approach	131	73 (55.7%)	110	47 (42.7%)	**0.044**
EIS as last endoscopic variceal therapeutic approach	131	10 (7.6%)	110	10 (9.1%)	0.683
Interval between last endoscopic variceal therapy and CT (days)	83	322.35 ± 399.43 188.00 (1.00–1644.00)	53	303.47 ± 328.70 201.00 (3.00–1676.00)	0.598
NSBBs within 1 month before admission	111	15 (13.5%)	79	16 (20.3%)	0.215

Laboratory data					
Red blood cell (10^12^/L)	131	3.64 ± 0.96 3.70 (1.59–9.92)	110	3.60 ± 0.82 3.76 (1.51–5.05)	0.964
Hemoglobin (g/L)	131	100.87 ± 27.68 101.00 (28.00–181.00)	110	105.80 ± 29.28 106.00 (32.00–159.00)	0.152
White blood cell (10^9^/L)	131	3.73 ± 2.90 3.20 (0.70–21.60)	110	4.80 ± 3.03 4.30 (1.00–20.80)	**<0.0001**
Platelet (10^9^/L)	131	86.65 ± 76.13 68.00 (15.00–681.00)	110	120.42 ± 85.34 91.00 (23.00–470.00)	**<0.0001**
Total bilirubin (*µ*mol/L)	131	26.14 ± 25.71 18.50 (5.60–215.30)	110	26.45 ± 27.96 17.60 (6.20–216.50)	0.614
Albumin (g/L)	131	34.77 ± 6.47 35.30 (14.20–71.40)	110	34.55 ± 5.97 35.15 (19.00–50.60)	0.766
Alanine aminotransferase (U/L)	131	25.77 ± 15.90 20.95 (4.23–99.13)	110	33.98 ± 39.01 24.33 (4.47–332.50)	0.119
Aspartate aminotransferase (U/L)	131	37.47 ± 24.15 28.62 (9.63–151.35)	110	45.59 ± 43.11 32.52 (9.74–376.35)	0.057
Alkaline phosphatase (U/L)	131	100.08 ± 62.67 84.94 (24.35–399.34)	110	124.22 ± 109.93 94.61 (31.00–983.93)	**0.014**
*γ*-Glutamyl transpeptidase (U/L)	131	76.67 ± 193.57 28.60 (9.64–1779.18)	110	107.93 ± 234.39 41.05 (7.49–1680.03)	**0.042**
Blood urea nitrogen (mmol/L)	131	5.65 ± 2.48 5.16 (1.88–20.15)	110	5.82 ± 2.77 5.29 (1.86–18.83)	0.897
Creatinine (*µ*mol/L)	131	65.57 ± 18.07 62.30 (36.39–178.55)	110	62.74 ± 16.30 58.67 (27.95–112.58)	0.223
Potassium (mmol/L)	131	3.90 ± 0.42 3.91 (2.70–5.87)	110	3.87 ± 0.43 3.97 (2.42–4.96)	0.950
Sodium (mmol/L)	131	138.72 ± 2.62 139.00 (127.00–147.70)	110	138.84 ± 3.65 139.55 (118.00–145.20)	0.194
Prothrombin time (seconds)	131	16.52 ± 2.17 16.20 (12.50–23.10)	110	16.06 ± 2.85 15.20 (12.60–28.00)	**0.005**
Activated partial thromboplastin time (seconds)	131	40.86 ± 5.01 40.20 (30.30–58.10)	110	40.39 ± 6.17 39.90 (19.80–71.30)	0.400
International normalized ratio	131	1.35 ± 0.25 1.31 (1.01–2.56)	110	1.30 ± 0.31 1.22 (0.94–2.77)	**0.005**
Child-Pugh score	131	6.77 ± 1.61 6.00 (5.00–12.00)	110	6.69 ± 1.71 6.00 (5.00–13.00)	0.534
Child-Pugh class (A/B/C)	131	71 (54.2%)/51 (38.9%)/9 (6.9%)	110	60 (54.5%)/42 (38.2%)/8 (7.3%)	0.988
MELD score	131	6.93 ± 4.21 6.66 (0.03–24.73)	110	5.93 ± 4.77 5.09 (−2.13–27.42)	**0.017**
EVs on endoscopy^b^	110	103 (93.6%)	99	72 (72.7%)	**<0.0001**
EVNTs on endoscopy^b^	109^c^	65 (59.6%)	99	39 (39.4%)	**0.002**

**Notes:** ^a^ECVs could not be evaluated because the venous vessels were not obviously enhanced in 2 patients;^b^as for the patients who underwent endoscopic variceal therapy, only EVs on endoscopy performed after CT during the same hospitalizations were evaluated; as for the patients who did not undergo endoscopic variceal therapy, EVs on endoscopy performed during the same hospitalizations were evaluated, regardless of the order of CT and endoscopy;^c^EVNTs could not be evaluated due to the absence of detailed grade of EVs in their endoscopic reports. Pts, patients; SD, standard deviation; EVL, endoscopic variceal ligation; EIS, endoscopic injection sclerotherapy; CT, computed tomography; NSBBs, nonselective beta-blockers; MELD, model for end stage of liver disease; EVs, esophageal varices; ECVs, esophageal collateral veins.

**Table 3:** Univariate and multivariate analysis for risk factors for ECVs.

Variables	No. Pts^a^	Univariate analysis			Multivariate analysis		
		Odds ratio	95% CI	*p* value	Odds ratio	95% CI	*p* value
Age (Years)	241	1.010	0.985–1.035	0.436			
Sex (male)	241	1.227	0.701–2.148	0.474			

Etiology of liver diseases							
Hepatitis B virus infection	241	1.411	0.843–2.363	0.191			
Hepatitis C virus infection	241	0.272	0.095–0.782	**0.016**	0.250	0.074–0.846	**0.026**
Alcohol abuse	241	1.152	0.683–1.943	0.596			
Drug related	241	1.029	0.410–2.581	0.952			
Autoimmune liver diseases	241	0.634	0.228–1.760	0.381			

Clinical presentations at admission							
Hepatic encephalopathy	241	0.204	0.022–1.851	0.158			
Gastrointestinal bleeding	241	1.415	0.838–2.388	0.194			
Ascites	241	1.479	0.887–2.464	0.133			

History							
History of gastrointestinal bleeding	241	1.164	0.681–1.989	0.597			
History of endoscopic variceal therapy	241	1.608	0.960–2.693	**0.071**			
EVL alone as last endoscopic variceal therapeutic approach	241	1.687	1.012–2.814	**0.045**	1.929	1.016–3.661	**0.044**
EIS alone as last endoscopic variceal therapeutic approach	241	0.826	0.331–2.065	0.683			
Interval between last endoscopic variceal therapy and CT (days)	136	1.000	0.999–1.001	0.772			
NSBBs within 1 month before admission	190	0.615	0.284–1.332	0.218			

Laboratory data							
Red blood cell (10^12^/L)	241	1.060	0.798–1.408	0.687			
Hemoglobin (g/L)	241	0.994	0.985–1.003	0.181			
White blood cell (10^9^/L)	241	0.876	0.793–0.968	**0.010**			
Platelet (10^9^/L)	241	0.994	0.990–0.998	**0.003**	0.993	0.988–0.998	**0.008**
Total bilirubin (*µ*mol/L)	241	1.000	0.990–1.009	0.928			
Albumin (g/L)	241	1.006	0.966–1.048	0.777			
Alanine aminotransferase (U/L)	241	0.987	0.975–1.000	**0.043**	0.989	0.973–1.004	0.152
Aspartate aminotransferase (U/L)	241	0.992	0.983–1.001	**0.084**			
Alkaline phosphatase (U/L)	241	0.996	0.992–1.000	**0.047**	1.000	0.996–1.004	0.966
*γ*-Glutamyl transpeptidase (U/L)	241	0.999	0.998–1.001	0.274			
Blood urea nitrogen (mmol/L)	241	0.976	0.886–1.075	0.623			
Creatinine (*µ*mol/L)	241	1.010	0.994–1.026	0.209			
Potassium (mmol/L)	241	1.227	0.670–2.244	0.508			
Sodium (mmol/L)	241	0.988	0.910–1.072	0.763			
Prothrombin time (seconds)	241	1.078	0.970–1.198	0.162			
Activated partial thromboplastin time (seconds)	241	1.015	0.970–1.063	0.517			
International normalized ratio	241	2.007	0.765–5.262	0.157			
Child-Pugh score	241	1.030	0.883–1.202	0.707			
MELD score	241	1.053	0.992–1.117	**0.088**	1.061	0.987–1.140	0.110
EVs on endoscopy^b^	209	5.518	2.279–13.358	**<0.0001**			
EVNTs on endoscopy^b^	208^c^	2.273	1.304–3.962	**0.004**	2.422	1.297–4.522	**0.006**

**Notes:** ^a^ECVs could not be evaluated because the venous vessels were not obviously enhanced in 2 patients; ^b^as for the patients who underwent endoscopic variceal therapy, only EVs on endoscopy performed after CT during the same hospitalizations were evaluated; as for the patients who did not undergo endoscopic variceal therapy, EVs on endoscopy performed during the same hospitalizations were evaluated, regardless of the order of CT and endoscopy; ^c^EVNTs could not be evaluated due to the absence of detailed grade of EVs in their endoscopic reports. CI, confidence interval; EVL, endoscopic variceal ligation; EIS, endoscopic injection sclerotherapy; CT, computed tomography; NSBBs, nonselective beta-blockers; MELD, model for end stage of liver disease; EVs, esophageal varices; ECVs, esophageal collateral veins.

**Table 4:** Comparison of patients with previous endoscopic variceal therapy between ECVs and no ECVs groups.

Variables	ECVs		No ECVs		*p* value
	No. Pts^a^	Mean ± SD Median (range) or frequency (percentage)	No. Pts^a^	Mean ± SD Median (range) or frequency (percentage)	
Age (Years)	83	57.38 ± 10.38	57	54.44 ± 10.53	0.150
Sex (male)	83	64 (77.1%)	57	36 (63.2%)	0.073

Etiology of liver diseases					
Hepatitis B virus infection	83	44 (53.0%)	57	23 (40.4%)	0.141
Hepatitis C virus infection	83	2 (2.4%)	57	9 (15.8%)	**0.010**
Alcohol abuse	83	31 (37.3%)	57	17 (29.8%)	0.357
Drug related	83	3 (3.6%)	57	4 (7.0%)	0.608
Autoimmune liver diseases	83	4 (4.8%)	57	7 (12.3%)	0.196

Clinical presentations at admission					
Hepatic encephalopathy	83	0 (0.0%)	57	2 (3.5%)	0.164
Gastrointestinal bleeding	83	32 (38.6%)	57	15 (26.3%)	0.132
Ascites (no/mild/moderate-severe)	83	36 (43.4%)/36 (43.4%)/11 (13.3%)	57	27 (47.4%)/23 (40.4%)/7 (12.3%)	0.897

History					
History of gastrointestinal bleeding	83	72 (86.7%)	57	55 (96.5%)	0.051
EVL as last endoscopic variceal therapeutic approach	83	73 (88.0%)	57	47 (82.5%)	0.361
EIS as last endoscopic variceal therapeutic approach	83	10 (12.0%)	57	10 (17.5%)	0.361
Interval between last endoscopic variceal therapy and CT (Days)	83	322.35 ± 399.43 188.00 (1.00–1644.00)	53	303.47 ± 328.70 201.00 (3.00–1676.00)	0.598
NSBBs within 1 month before admission	76	15 (19.7%)	43	15 (34.9%)	0.068

Laboratory data					
Red blood cell (10^12^/L)	83	3.70 ± 0.80 3.78 (1.77–5.49)	57	3.69 ± 0.78 3.84 (1.51–4.94)	0.973
Hemoglobin (g/L)	83	102.88 ± 25.40 104.00 (46.00–161.00)	57	104.30 ± 26.21 106.00 (32.00–153.00)	0.722
White blood cell (10^9^/L)	83	3.54 ± 1.95 3.30 (1.20–11.90)	57	4.43 ± 2.65 3.80 (1.30–15.20)	**0.036**
Platelet (10^9^/L)	83	86.96 ± 64.64 68.00 (15.00–457.00)	57	115.98 ± 80.36 86.00 (23.00–448.00)	**0.014**
Total bilirubin (*µ*mol/L)	83	22.00 ± 13.42 17.10 (7.60–78.20)	57	21.99 ± 14.41 17.60 (8.80–92.60)	0.916
Albumin (g/L)	83	35.41 ± 6.59 35.60 (21.50–71.40)	57	34.95 ± 4.95 35.20 (23.10–45.60)	0.620
Alanine aminotransferase (U/L)	83	22.97 ± 10.76 20.95 (6.78–54.73)	57	27.21 ± 18.56 21.93 (4.52–113.78)	0.346
Aspartate aminotransferase (U/L)	83	33.51 ± 19.23 27.81 (9.63–130.22)	57	37.23 ± 18.36 31.28 (17.22–118.28)	**0.045**
Alkaline phosphatase (U/L)	83	88.02 ± 49.41 79.00 (24.35–351.33)	57	120.61 ± 68.41 98.96 (45.45–466.34)	**<0.0001**
*γ*-Glutamyl transpeptidase (U/L)	83	44.40 ± 54.54 23.49 (9.64–357.32)	57	84.54 ± 221.41 39.14 (7.49–1680.03)	**0.008**
Blood urea nitrogen (mmol/L)	83	5.93 ± 2.87 5.33 (1.88–20.15)	57	5.81 ± 2.43 5.28 (1.88–14.69)	0.966
Creatinine (*µ*mol/L)	83	64.26 ± 12.94 62.12 (36.39–108.80)	57	58.78 ± 14.12 56.99 (34.51–109.21)	**0.010**
Potassium (mmol/L)	83	3.92 ± 0.37 3.86 (3.34–5.87)	57	3.94 ± 0.42 3.99 (2.76–4.96)	0.289
Sodium (mmol/L)	83	138.66 ± 2.11 138.70 (133.40–147.70)	57	138.78 ± 3.41 139.50 (127.50–143.80)	0.087
Prothrombin time (seconds)	83	16.23 ± 1.84 16.10 (13.50–22.50)	57	15.97 ± 2.32 15.40 (12.80–25.20)	0.160
Activated partial thromboplastin time (seconds)	83	40.13 ± 4.42 39.70 (33.30–54.10)	57	40.58 ± 5.93 39.60 (32.80–71.30)	0.966
International normalized ratio	83	1.33 ± 0.23 1.30 (1.06–2.56)	57	1.30 ± 0.25 1.23 (0.98–2.41)	0.212
Child-Pugh score	83	6.51 ± 1.27 6.00 (5.00–11.00)	57	6.47 ± 1.35 6.00 (5.00–11.00)	0.809
Child-Pugh class (A/B/C)	83	48 (57.8%)/34 (41.0%)/1 (1.2%)	57	32 (56.1%)/22 (38.6%)/3 (5.3%)	0.366
MELD score	83	6.33 ± 3.51 6.66 (0.48–15.42)	57	5.15 ± 4.24 4.28 (−1.32–19.38)	**0.018**
EVs on endoscopy^b^	62	57 (91.9%)	46	39 (84.8%)	0.242
EVNTs on endoscopy^b^	62	27 (43.5%)	46	12 (26.1%)	0.062

Notes: ^a^ECVs could not be evaluated because the venous vessels were not obviously enhanced in 1 patient; ^b^as for the patients who underwent endoscopic variceal therapy, only EVs on endoscopy performed after CT during the same hospitalizations were evaluated; as for the patients who did not undergo endoscopic variceal therapy, EVs on endoscopy performed during the same hospitalizations were evaluated, regardless of the order of CT and endoscopy. Pts, patients; SD, standard deviation; EVL, endoscopic variceal ligation; EIS, endoscopic injection sclerotherapy; CT, computed tomography; NSBBs, nonselective beta-blockers; MELD, model for end stage of liver disease; EVs, esophageal varices; ECVs, esophageal collateral veins.

**Table 5:** Univariate and multivariate analysis for risk factors for ECVs in patients with previous endoscopic variceal therapy.

Variables	No. Pts^a^	Univariate analysis			Multivariate analysis		
		Odds ratio	95% CI	*p* value	Odds ratio	95% CI	*p* value
Age (years)	140	1.028	0.994–1.062	0.107			
Sex (male)	140	1.965	0.935–4.130	**0.075**	1.171	0.312–4.391	0.815

Etiology of liver diseases							
Hepatitis B virus infection	140	1.668	0.843–3.300	0.142			
Hepatitis C virus infection	140	0.132	0.027–0.635	**0.012**	NA	NA	0.999
Alcohol abuse	140	1.403	0.682–2.885	0.358			
Drug related	140	0.497	0.107–2.310	0.372			
Autoimmune liver diseases	140	0.362	0.101–1.299	0.119			

Clinical presentations at admission							
Hepatic encephalopathy	140	NA	NA	0.999			
Gastrointestinal bleeding	140	1.757	0.841–3.671	0.134			
Ascites	140	1.175	0.597–2.313	0.641			

History							
History of gastrointestinal bleeding	140	0.238	0.051–1.118	**0.069**	NA	NA	0.999
EVL alone as last endoscopic variceal therapeutic approach	140	1.553	0.601–4.016	0.364			
EIS alone as last endoscopic variceal therapeutic approach	140	0.644	0.249–1.665	0.364			
Interval between last endoscopic variceal therapy and CT (days)	136	1.000	0.999–1.001	0.772			
NSBBs within 1 month before admission	119	0.459	0.197–1.068	**0.071**	0.294	0.091–0.957	**0.042**

Laboratory data							
Red blood cell (10^12^/L)	140	1.028	0.670–1.578	0.899			
Hemoglobin (g/L)	140	0.998	0.985–1.011	0.747			
White blood cell (10^9^/L)	140	0.840	0.717–0.984	**0.031**			
Platelet (10^9^/L)	140	0.994	0.989–0.999	**0.027**	0.993	0.983–1.003	0.157
Total bilirubin (*µ*mol/L)	140	1.000	0.976–1.025	0.997			
Albumin (g/L)	140	1.013	0.956–1.073	0.654			
Alanine aminotransferase (U/L)	140	0.979	0.955–1.004	0.102			
Aspartate aminotransferase (U/L)	140	0.990	0.972–1.008	0.258			
Alkaline phosphatase (U/L)	140	0.989	0.982–0.997	**0.004**	0.997	0.989–1.006	0.536
*γ*-Glutamyl transpeptidase (U/L)	140	0.996	0.990–1.002	0.204			
Blood urea nitrogen (mmol/L)	140	1.018	0.896–1.156	0.784			
Creatinine (*µ*mol/L)	140	1.033	1.005–1.062	**0.022**			
Potassium (mmol/L)	140	0.867	0.361–2.080	0.749			
Sodium (mmol/L)	140	0.983	0.867–1.115	0.794			
Prothrombin time (seconds)	140	1.066	0.899–1.264	0.461			
Activated partial thromboplastin time (seconds)	140	0.983	0.920–1.050	0.609			
International normalized ratio	140	1.725	0.392–7.590	0.471			
Child-Pugh score	140	1.020	0.785–1.324	0.885			
MELD score	140	1.088	0.990–1.196	**0.079**	1.232	0.982–1.544	0.071
EVs on endoscopy^b^	108	2.046	0.605–6.915	0.249			
EVNTs on endoscopy^b^	108	2.186	0.955–5.001	**0.064**	2.931	0.879–9.780	0.080

**Notes:** ^a^ECVs could not be evaluated because the venous vessels were not obviously enhanced in 1 patient; ^b^as for the patients who underwent endoscopic variceal therapy, only EVs on endoscopy performed after CT during the same hospitalizations were evaluated; as for the patients who did not undergo endoscopic variceal therapy, EVs on endoscopy performed during the same hospitalizations were evaluated, regardless of the order of CT and endoscopy. CI, confidence interval; EVL, endoscopic variceal ligation; EIS, endoscopic injection sclerotherapy; CT, computed tomography; NSBBs, nonselective beta-blockers; MELD, model for end stage of liver disease; EVs, esophageal varices; ECVs, esophageal collateral veins; NA, not available.

**Table 6:** Comparison of patients without previous endoscopic variceal therapy between ECVs and no ECVs groups.

Variables	ECVs		No ECVs		*p* value
	No. Pts^a^	Mean ± SD Median (range) or frequency (percentage)	No. Pts^a^	Mean ± SD Median (range) or frequency (percentage)	
Age (Years)	48	52.52 ± 10.88	53	54.67 ± 9.51	0.348
Sex (male)	48	32 (66.7%)	53	40 (75.5%)	0.329

Etiology of liver diseases					
Hepatitis B virus infection	48	17 (35.4%)	53	19 (35.8%)	0.964
Hepatitis C virus infection	48	3 (6.2%)	53	5 (9.4%)	0.824
Alcohol abuse	48	21 (43.8%)	53	23 (4.34%)	0.971
Drug related	48	8 (16.7%)	53	5 (9.4%)	0.278
Autoimmune liver diseases	48	3 (6.2%)	53	2 (3.8%)	0.909

Clinical presentations at admission					
Hepatic encephalopathy	48	1 (2.1%)	53	2 (3.8%)	1.000
Gastrointestinal bleeding	48	24 (50.0%)	53	23 (43.4%)	0.506
Ascites (no/mild/moderate-severe)	48	18 (37.5%)/16 (33.3%)/14 (29.2%)	53	29 (54.7%)/14 (26.4%)/10 (18.9%)	0.209

History					
History of gastrointestinal bleeding	48	17 (35.4%)	53	16 (30.2%)	0.576
NSBBs within 1 month before admission	35	0 (0.0%)	36	1 (2.8%)	1.000

Laboratory data					
Red blood cell (10^12^/L)	48	3.56 ± 1.20 3.51 (1.59–9.92)	53	3.51 ± 0.87 3.71 (1.91–5.05)	0.897
Hemoglobin (g/L)	48	97.40 ± 31.20 96.00 (28.00–181.00)	53	107.42 ± 32.45 106.00 (37.00–159.00)	0.103
White blood cell (10^9^/L)	48	4.05 ± 4.06 3.15 (0.70–21.60)	53	5.19 ± 3.38 4.5.0 (1.00–20.80)	**0.002**
Platelet (10^9^/L)	48	86.10 ± 93.50 66.50 (26.00–681.00)	53	125.19 ± 90.92 98.00 (30.00–470.00)	**0.003**
Total bilirubin (*µ*mol/L)	48	33.30 ± 37.83 20.95 (5.60–215.30)	53	31.24 ± 37.02 19.30 (6.20–216.50)	0.324
Albumin (g/L)	48	33.68 ± 6.16 33.65 (14.20–45.10)	53	34.11 ± 6.92 34.60 (19.00–50.60)	0.916
Alanine aminotransferase (U/L)	48	30.61 ± 21.43 21.24 (4.23–99.13)	53	41.26 ± 52.10 28.57 (4.47–332.50)	0.395
Aspartate aminotransferase (U/L)	48	44.33 ± 29.87 32.20 (15.35–151.35)	53	54.57 ± 58.09 38.96 (8.74–376.35)	0.589
Alkaline phosphatase (U/L)	48	120.94 ± 76.83 94.56 (33.00–399.34)	53	128.10 ± 142.35 83.00 (31.00–983.93)	0.395
*γ*-Glutamyl transpeptidase (U/L)	48	132.48 ± 305.66 41.52 (11.42–1779.18)	53	133.09 ± 247.23 41.56 (8.23–1283.03)	0.903
Blood urea nitrogen (mmol/L)	48	5.16 ± 1.52 4.99 (2.31–9.53)	53	5.83 ± 3.12 5.29 (1.86–18.83)	0.799
Creatinine (*µ*mol/L)	48	67.84 ± 24.56 63.03 (37.66–178.55)	53	66.99 ± 17.50 64.75 (27.95–112.58)	0.572
Potassium (mmol/L)	48	3.87 ± 0.50 3.94 (2.70–5.19)	53	3.79 ± 0.43 3.95 (2.42–4.64)	0.452
Sodium (mmol/L)	48	138.84 ± 3.35 139.75 (127.00–143.40)	53	138.92 ± 3.92 139.60 (118.00–145.20)	0.984
Prothrombin time (seconds)	48	17.03 ± 2.60 16.45 (12.50–23.10)	53	16.17 ± 3.34 15.20 (12.60–28.00)	**0.010**
Activated partial thromboplastin time (seconds)	48	42.13 ± 5.72 41.90 (30.30–58.10)	53	40.20 ± 6.47 40.10 (19.80–55.30)	0.134
International normalized ratio	48	1.40 ± 0.27 1.33 (1.01–2.07)	53	1.31 ± 0.36 1.20 (0.94–2.77)	**0.007**
Child-Pugh score	48	7.23 ± 1.99 7.00 (5.00–12.00)	53	6.92 ± 2.02 6.00 (5.00–13.00)	0.350
Child-Pugh class (A/B/C)	48	23 (47.9%)/17 (35.4%)/8 (16.75)	53	28 (52.8%)/20 (37.7%)/5 (9.4%)	0.554
MELD score	48	7.99 ± 5.09 6.72 (0.03–24.73)	53	6.78 ± 5.19 6.09 (−2.13–27.42)	0.163
EVs on endoscopy	48	46 (95.8%)	53	33 (62.3%)	**<0.0001**
EVNTs on endoscopy	47^b^	39 (80.9%)	53	27 (50.9%)	**0.002**

**Notes:** ^a^ECVs could not be evaluated because the venous vessels were not obviously enhanced in 1 patient; ^b^EVNTs could not be evaluated due to the absence of detailed grade of EVs in their endoscopic reports. Pts, patients; SD, standard deviation; NSBBs, nonselective beta-blockers; MELD, model for end stage of liver disease; EVs, esophageal varices; ECVs, esophageal collateral veins.

**Table 7:** Univariate and multivariate analysis for risk factors for ECVs in patients without previous endoscopic variceal therapy.

Variables	No. Pts^a^	Univariate analysis			Multivariate analysis		
		Odds ratio	95% CI	*p* value	Odds ratio	95% CI	*p* value
Age (years)	101	0.979	0.941–1.018	0.291			
Sex (male)	101	1.538	0.646–3.661	0.330			

Etiology of liver diseases							
Hepatitis B virus infection	101	0.981	0.434–2.218	0.964			
Hepatitis C virus infection	101	0.640	0.145–2.834	0.557			
Alcohol abuse	101	1.014	0.462–2.230	0.971			
Drug related	101	1.920	0.582–6.334	0.284			
Autoimmune liver diseases	101	1.700	0.272–10.635	0.571			

Clinical presentations at admission							
Hepatic encephalopathy	101	0.543	0.048–6.181	0.622			
Gastrointestinal bleeding	101	1.304	0.595–2.858	0.507			
Ascites	101	2.014	0.908–4.465	**0.085**	1.488	0.628–3.527	0.367

History							
History of gastrointestinal bleeding	101	1.268	0.551–2.917	0.576			
NSBBs within 1 month before admission	71	NA	NA	1.000			

Laboratory data							
Red blood cell (10^12^/L)	101	1.042	0.712–1.526	0.832			
Hemoglobin (g/L)	101	0.99	0.978–1.003	0.119			
White blood cell (10^9^/L)	101	0.911	0.805–1.031	0.141			
Platelet (10^9^/L)	101	0.994	0.987–1.000	**0.054**	0.995	0.990–1.001	0.092
Total bilirubin (*µ*mol/L)	101	1.001	0.991–1.012	0.781			
Albumin (g/L)	101	0.990	0.932–1.051	0.737			
Alanine aminotransferase (U/L)	101	0.992	0.979–1.005	0.219			
Aspartate aminotransferase (U/L)	101	0.995	0.985–1.005	0.292			
Alkaline phosphatase (U/L)	101	0.999	0.996–1.003	0.756			
*γ*-Glutamyl transpeptidase (U/L)	101	1.000	0.999–1.001	0.991			
Blood urea nitrogen (mmol/L)	101	0.891	0.750–1.059	0.191			
Creatinine (*µ*mol/L)	101	1.002	0.983–1.021	0.839			
Potassium (mmol/L)	101	1.505	0.635–3.564	0.353			
Sodium (mmol/L)	101	0.994	0.892–1.107	0.912			
Prothrombin time (seconds)	101	1.102	0.963–1.262	0.158			
Activated partial thromboplastin time (seconds)	101	1.054	0.986–1.127	0.121			
International normalized ratio	101	2.543	0.705–9.174	0.154			
Child-Pugh score	101	1.080	0.887–1.315	0.444			
MELD score	101	1.048	0.969–1.134	0.243			
EVs on endoscopy	101	13.939	3.046–63.783	**0.001**			
EVNTs on endoscopy	100^b^	4.066	1.646–10.044	**0.002**	3.714	1.469–9.391	**0.006**

Notes: ^a^ECVs could not be evaluated because the venous vessels were not obviously enhanced in 1 patient;^b^EVNTs could not be evaluated due to the absence of detailed grade of EVs in their endoscopic reports. CI, confidence interval; NSBBs, nonselective beta-blockers; MELD, model for end stage of liver disease; EVs, esophageal varices; ECVs, esophageal collateral veins.

## Data Availability

The data used to support the findings of this study are available from the corresponding author upon request.

## Abbreviations

EVs: Esophageal varices
EVL: Endoscopic variceal ligation
EIS: Endoscopic injection sclerotherapy
ECVs: Esophageal collateral veins
CT: Computed tomography
HE: Hepatic encephalopathy
GIB: Gastrointestinal bleeding
WBC: White blood cell
PLT: Platelet
TBIL: Total bilirubin
ALB: Albumin
AKP: Alkaline phosphatase
GGT: *γ*-Glutamine transferase
Cr: Creatinine
PT: Prothrombin time
INR: International normalized ratio
NSBBs: Nonselective beta-blockers
MELD: Model for end stage of liver disease
EVNTs: Esophageal varices needing treatment
RC: Red color
ORs: Odds ratios
CIs: Confidence intervals
HCV: Hepatitis C virus
para-EVs: Para-esophageal veins
peri-EVs: Peri-esophageal veins
PVs: Perforating veins
PSR: Platelet count to spleen diameter ratio.
